# Pain Experiences and Their Relation to Opioid Misuse Risk and Emotion Dysregulation

**DOI:** 10.1155/2020/7234625

**Published:** 2020-11-11

**Authors:** Jonathan W. Nauser, Cecelia I. Nelson, Richard T. Gross, Alison M. Vargovich

**Affiliations:** ^1^West Virginia University, Morgantown, WV 26506, USA; ^2^University at Buffalo, SUNY, Buffalo, NY 14260, USA

## Abstract

Pain is a complex, multidimensional experience but often is measured as a unidimensional experience. This study aimed to separately assess the sensory and affective components of pain and identify their relations to important pain-related outcomes, particularly in terms of opioid misuse risk and emotion dysregulation among patients with chronic pain receiving treatment in Appalachia. Two hundred and twelve patients presenting to a multidisciplinary pain center completed the Difficulties in Emotion Regulation Scale (DERS-18), Screener and Opioid Assessment for Patients with Pain—Revised (SOAPP-R), and short-form McGill Pain Questionnaire (SF-MPQ). The sensory experience of pain was unrelated to emotion dysregulation (*r* = 0.06, *p* = 0.57) and weakly related to opioid misuse risk (*r* = 0.182, *p* < 0.05). In contrast, the affective experience of pain was moderately related to emotion dysregulation (*r* = 0.217, *p* < 0.05) and strongly related to opioid misuse risk (*r* = 0.37, *p* < 0.01). In addition, emotion dysregulation predicted variance in opioid misuse risk above and beyond the affective and sensory experiences of pain ((*b* = 0.693, *p* < 0.001). The results suggest patients with a strong affective experience versus sensory experience of pain and challenges with emotion regulation may require a more comprehensive intervention to address these underlying components in order to reduce their risk of misusing opioid medications.

## 1. Introduction

Chronic pain is understood to be a complex biopsychosocial experience [1]; however, measurement of pain in many clinical settings fails to differentiate between these symptom domains. Instead, pain is reduced to a primarily sensory event of varying intensity. Failure to recognize and address this complexity contributes to a myriad of problems associated with chronic pain, from untreated pain-related distress to more macroconcerns, such as the opioid epidemic [2]. To remedy this issue, pain researchers across disciplines, including Ballantyne and Sullivan [3], have proposed utilizing multiple measures of pain-related factors to clarify a person's pain experience and to further inform possible multimodal treatments for chronic pain. Measuring patients' chronic pain experience requires assessment that acknowledges the various qualitative differences in pain and goes beyond simple pain severity [4–6]. Patients also report that a numeric rating scale alone does not capture their subjective experience of pain despite its common use in medical settings [7].

When asked to quantify pain intensity, the validity of numerical scales is impacted by patient's inclusion of other concurrent sensations and experiences rather than simply pain intensity [8]. In fairness, it is difficult for patients to tease apart pain severity from the other aspects of pain that contribute to its discomfort in a single question. In addition, recent research suggests the three most common measures used to assess pain intensity (i.e., Visual Analog Scale, Numeric Rating Scale, and Pain Severity Subscale of the Brief Pain Inventory) demonstrate low- to very low-quality evidence of content validity, thus not providing particularly useful information to researchers and clinicians [9]. Alternatively, as an example, the McGill Pain Questionnaire (MPQ) and its short form (SF-MPQ) are widely used to assess both the sensory and affective components of pain, with an emphasis on the unique quality of the pain experience [10, 11]. Assessing these components offers critical information regarding the perceptual qualities of pain [4] and provides valuable insight into the patient's pain experience beyond the severity and duration of their pain. By including more comprehensive assessment of pain factors, the barriers to pain relief are more easily elucidated and appropriate treatment can be offered.

When addressing barriers to pain relief, emotion regulation issues may be overlooked, particularly in a traditional medical setting; however, these issues can significantly complicate a patient's ability to manage chronic pain and pain itself may lead to poorer emotion regulation. The experience of chronic pain is further complicated by the likely difficulty in regulation of affect associated with this chronic stressor. For example, suffering from chronic pain may present as a constant stressor that increases the amount of negative affect one experiences and thus it might become increasingly difficult for one to regulate their emotions [12]. Emotion dysregulation is defined as the absence of awareness and lack of acceptance of one's emotions, the inability to change emotions in accordance with one's goals, and the inability to control behavior in the face of negative emotions [13]. Like all coping strategies, there are positive/healthy and negative/unhealthy approaches to emotion regulation. Positive or healthy emotion regulation approaches include skills such as cognitive reappraisal, mindfulness, acceptance, and problem solving. Negative or unhealthy emotion regulation approaches include self-injury, avoidance, and substance misuse, and while these may be effective temporarily, they often lead to more problems. This point is supported by the fear-avoidance model of chronic pain, which outlines the ways in which emotion, fear specifically, can promote avoidance which in turn perpetuates disability and fear [14]. Emotion dysregulation in patients with chronic pain may lead to difficulty in accurately assessing pain intensity and other pain factors, negatively impacting pain perception [7–9]. Emotion dysregulation is associated with modifying experiences of pain. Ruiz-Aranda et al. [15] found that women with the emotion regulation strategy of emotional repair (i.e., attenuate to and value feelings, clarity about feelings, and utilization of positive thinking to repair negative moods) reported having less pain during an experimental pain induction. A lack of emotion regulation plays a role in the increase in negative affect and dysfunctional beliefs related to pain [16], while regulation of positive and negative affect predicts greater decreases in pain [17]. Hamilton et al. [18] also found the magnitude of pain reactivity varied as a function of both emotional intensity and ability to regulate emotion. Thus, evaluating emotion regulation abilities in patients with chronic pain may be helpful in understanding difficulties in managing pain and exacerbations in pain symptoms.

Examining emotion dysregulation more specifically in the chronic pain population reveals a link with opioid misuse risk and poor health-related outcomes [19, 20]. Furthermore, opioid misuse is independently associated with both chronic pain experience [21] and emotion dysregulation [22]. Thus, it is entirely possible that individuals who experience chronic pain may rely upon opioid medications in order to assist in emotion regulation. Garland et al. [21] noted that, in addition to attenuating the sensory aspects of pain, opioids may ameliorate the affective pain experience or patient's perception of pain. When uncontrolled use of opioids couples with chronic pain, there is dysregulation in the reward-processing center of the brain, which suggests that the affective experience of pain may drive the enduring and relapsing nature of substance use disorder, due to the way in which opioid medication stimulates this area of the brain [23]. This research suggests that although opioids are prescribed with the goal of alleviating nociceptive pain, or the sensory experience of pain, individuals may continue using opioid medication or misusing it due to its affective or emotional effects (i.e., an unhealthy emotion regulation strategy for chronic pain). Because of the potential misuse of prescribed opioid medication and its addictive properties, it is important to identify risk factors for misuse prior to prescribing [24, 25].

To date, limited research has investigated the affective and sensory experiences of chronic pain and their relation with opioid misuse and emotion dysregulation. The current study aims to examine pain as an affective and sensory experience, as measured by the SF-MPQ to identify associations between these pain experiences, opioid misuse risk, and emotion dysregulation, as well as to identify the factors that contribute to variance in opioid misuse risk to identify target areas of treatment in future research. The authors propose four hypotheses related to this study: (1) in support of prior findings, the affective experience of pain will be associated with opioid misuse risk; (2) given the previous associations identified between affect and opioid misuse, as well as pain and emotion dysregulation, emotion dysregulation will be associated with opioid misuse risk; (3) based upon prior research identifying emotional and affective components of pain as integral to opioid misuse, the sensory experience of pain will be unassociated with opioid misuse risk and emotion dysregulation; (4) lastly, given prior research, the affective experience of pain will predict variance in opioid misuse risk above and beyond the sensory experience of pain or emotion dysregulation.

## 2. Materials and Methods

This sample was part of a larger database from a previous study examining pain-related outcomes [26]. A sample of 212 patients with chronic pain presented to a multidisciplinary pain center at West Virginia University on referral from pain center physicians (52.8% female, mean age = 53.6, SD = 10.8). Patients participated in a psychological interview and completed a series of standardized measures as part of an evaluation to assess for risk of opioid misuse.

### 2.1. Measures

#### 2.1.1. Demographic Information

Data on patients' age, sex, education, duration of pain, and location of pain were gathered via self-report questionnaires (see Table 1 for demographics).

#### 2.1.2. Difficulties in Emotion Regulation Scale-18 (DERS-18)

The DERS-18 is a short-form version of the 36-item self-report measure of emotion dysregulation [13, 27]. Items are rated from 1 (“almost never”) to 5 (“almost always”), and some items are reverse coded. Higher scores indicate greater difficulty in emotion regulation. The scale consists of six subscales measuring differing types of difficulty in emotion regulation and demonstrates good reliability, internal consistency, and convergent validity, as well as concurrent validity with the original DERS [13]. None of the subscales of the DERS-18 were analyzed and solely total scores were utilized in this study due to the study's focus on general levels of emotional dysregulation in association with pain experience. Total scores range from 18 to 90, with greater scores indicating greater emotion dysregulation (i.e., poorer emotion regulation skills). The DERS-18 has an overall Cronbach's alpha of .91 [13]. The DERS-18 also demonstrated strong predictive validity in a diary study and strong concurrent validity with the original DERS scale [13].

#### 2.1.3. Short-Form McGill Pain Questionnaire (SF-MPQ)

The SF-MPQ is an abbreviated version of the McGill Pain Questionnaire [11], and it measures quality of pain [28] that utilizes 11 sensory descriptors and four affective descriptors (4) [11]. Items are rated by respondents from 0 (none) to 3 (severe). Scores range from 0 to 33 (Sensory Subscale) and 0 to 12 (Affective Subscale) with higher scores indicative of greater pain severity along with greater number of descriptors. The SF-MPQ also exhibited good sensitivity to change following chronic pain treatment [11]. The SF-MPQ demonstrated strong validity via significant correlations between subscale and total scores for the short form and long form [11].

#### 2.1.4. Screener and Opioid Assessment for Patients with Pain—Revised (SOAPP-R)

The SOAPP-R is a self-report measure that assesses risk for misuse of opioid medication [29]. The 24-item measure asks respondents to rate the frequency of occurrence using a Likert scale that ranges from 0 (“never”) to 4 (“very often”). Total scores range from 0 to 96, with higher scores indicating higher risk for opioid misuse. If the score is ≥18, this indicates high risk for opioid misuse. Good internal reliability, specificity, and sensitivity are demonstrated when using the SOAPP-R to identify those at elevated risk for opioid misuse [29]. Test-retest intraclass correlations for the total scale were 0.92. In addition, Cohen's *d* effect sizes were calculated for each item, and all were above 0.40 indicating strong predictive validity [29]. Cronbach's alpha for the total score was 0.88 demonstrating strong internal reliability [29].

## 3. Results and Discussion

To investigate the first three hypotheses, Pearson R correlations were calculated for the variables of interest (see Table 2). Correlations are interpreted such that a correlation of *r* = 0.1-0.2 is considered small, *r* = 0.2-0.3 is considered medium, and anything above *r* = 0.3 is considered large or strongly related [30]. The sensory and affective components of pain were uniquely related to the pain-related outcomes of opioid misuse risk and emotion dysregulation. In support of prior research and hypotheses, the affective experience of pain was strongly related to opioid misuse risk (*r* = 0.37, *p* < 0.01) and moderately related to emotion dysregulation (*r* = 0.217, *p* < 0.05). Contrary to hypotheses, the sensory experience of pain was related, though weakly, to opioid misuse risk (*r* = 0.182, *p* < 0.05), though unrelated to emotion dysregulation (*r* = 0.06, *p* = 0.57). For all correlations, see Table 2.

To investigate the fourth hypothesis, a multiple linear regression was conducted. Independent variables included the affective experience of pain, sensory experience of pain, and emotion dysregulation. The overall model significantly predicted variance in opioid misuse risk (*R* = 0.75, *R*^2^ = 0.57, *F* (3, 70) = 29.07, *p* < 0.001). Interestingly, and contrary to hypotheses, emotion dysregulation was the only predictor that emerged significant in the model (*b* = 0.693, *p* < 0.001), despite the prior associations between affective experience of pain and emotion dysregulation. Based upon Ferguson's [31] guidance on effect size in social science, this effect produced a medium effect size. For regression results, see Table 3.

Patients with a higher affective experience of pain may be at increased risk for emotion dysregulation or opioid misuse relative to those who have a stronger sensory experience of pain. Pain patients should routinely be interviewed about the nature of their pain and both biological and psychological factors that contribute to their experience. In support of these findings, recent research has identified emotional dysregulation as a key component of opioid misuse and suicidality in chronic pain patients [32]. The results of this study suggest that emotional dysregulation also plays a key role in opioid misuse risk, above and beyond the affective or sensory experiences of pain. Thus, future research may address how emotion dysregulation might be assessed or ameliorated in chronic pain patients prior to or concurrent with opioid treatment.

The study was limited in that it was correlational in nature; however, these findings have interesting implications for the treatment and future study of the biopsychosocial nature of pain and co-occurring substance use disorder. In addition, given that this study was cross-sectional in nature, no conclusions about the directionality of the relation identified in the multiple linear regression may be made. This study also relied upon self-report of all variables. Future studies might utilize more objective measures or indicators of pain or opioid use (rather than risk) in order to obtain multiple reports. To that point, it is imperative that future research addresses the biological portion of the biopsychosocial model in the relation between pain experience and opioid misuse and risk. It is entirely possible that endogenous opioids or genetic influence may have an impact on the relations identified in this study. Our results suggest that future research should investigate the possibility of a causal relation between the affective experiences of pain and opioid misuse and emotion dysregulation and potential nonopioid treatments for patients with a highly affective pain experience.

## 4. Conclusion

This study highlights the importance of assessing for pain experience and possible biological and psychological factors that contribute to chronic pain patients' experience. The sensory experience of pain was weakly associated with opioid misuse risk, but unrelated to emotion dysregulation. The affective experience of pain was related to opioid misuse risk and emotion dysregulation. Further research is needed to investigate a causal relationship between the affective and sensory experiences of pain, opioid misuse, and emotion dysregulation.

## Figures and Tables

**Table 1:** Demographic information.

Variable	Mean	SD	Frequency (%)
1. Age (years)	53.59	10.82	—
2. Education (years)	12.59	2.5	—
3. Pain duration (years)	12.32	10.18	—
4. Pain location (percent yes)			
Neck	—	—	41.60
Low back	—	—	83.80
Upper extremities	—	—	29.60
Lower extremities	—	—	61.40
Others	—	—	54.80

Percentages are each out of 100%, as participants often indicated multiple locations of pain.

**Table 2:** Summary of correlations, means, and standard deviations for main variables of interest.

Measure	1	2	3	4
1. SOAPP-R	—	0.69^*∗∗*^	0.182^*∗*^	0.367^*∗∗*^
2. DERS	0.69^*∗∗*^	—	0.06	0.217^*∗*^
3. Sensory	0.182^*∗*^	0.06	—	0.66^*∗∗*^
4. Affective	0.37^*∗∗*^	0.217^*∗*^	0.66^*∗∗*^	—
Mean	14.11	35.67	15.47	3.2
SD	9.29	12.19	7.09	3.15

^*∗*^Significance at *p* < 0.05; ^*∗∗*^significance at *p* < 0.01.

**Table 3:** Multiple linear regression.

Variable	*B*	Std. error	*B*	*T*
Affective	0.38	0.29	0.147	1.313
Sensory	0.109	0.131	0.091	0.831
Emotion dysregulation	0.483	0.059	0.693^*∗∗*^	8.256

^*∗∗*^ *p* < 0.001.

## Data Availability

The quantitative data used to support the findings of this study are available from the corresponding author upon request.
